# Ultra-Sensitive C-Reactive Protein (US-CRP) in Patients With Periodontal Disease and Risk of Acute Myocardial Infarction

**DOI:** 10.4021/cr11e

**Published:** 2011-01-20

**Authors:** Catalina Latorre Uriza, Francina Escobar Arregoces, Juliana Velosa Porras, Maria Beatriz Ferro Camargo, Alvaro Ruiz Morales

**Affiliations:** a Periodontal Department, Faculty of Dentistry, Pontificia Universidad Javeriana, Colombia; b Graduate Periodontics Program, Faculty of Dentistry, Pontificia Universidad Javeriana, Colombia; c Faculty of Dentistry, Pontificia Universidad Javeriana, Colombia; d Department of Internal Medicine and Department of Clinical Epidemiology, Faculty of Medicine, Pontificia Universidad Javeriana, Colombia

**Keywords:** Periodontal disease, Cardiovascular diseases, Ultra-sensitive C-reactive protein, Acute myocardial infarction, Smoking, Diabetes, Dyslipidemia

## Abstract

**Background:**

The purpose of this study was to determine if the US-CRP values associated with periodontal disease are risk markers for Acute Myocardial Infarction (AMI) and to determine if the US-CRP levels associated with recent AMI are higher in patients with Periodontal disease.

**Methods:**

In order to meet the goal of the study, a case control study design was conducted. The analysis sample consisted of 401 adults (30 - 75 years old), living in Bogota D.C., Colombia, from the Hospital Universitario San Ignacio, the Faculty of Dentistry at the Pontificia Universidad Javeriana, and the Fundacion Cardio Infantil. Patients with current infections, antibiotic use in the last 3 months, periodontal treatment at least six months before the baseline of this study, mouth ulcerations caused by any type of prosthesis, candidiasis, stomatitis, or less than 7 teeth in mouth were excluded. Periodontal examination for the case group and the control group was conducted by three previously calibrated examiners. Periodontal disease was diagnosed by the presence of bleeding on probing and attachment loss. The Chronic Periodontitis diagnosis was confirmed with these clinical signs, according to the 1999 Armitage classification. The assessment of the US-CRP was performed using the IMMULITE method containing one monoclonal and one polyclonal anti-CRP antibody. This method provides a measurement range of 0.1 - 500 mg/L. Statistical analysis of variables was performed with OR and confidence intervals. A multivariate analysis was performed to determine the association between the US-CRP increase, periodontal disease and acute myocardial infarction, adjusting for smoking and other confounding factors identified in the analysis.

**Results:**

The study population was constituted by 401 patients, 56.1% (225) males, with a mean age of 52.6. When groups were compared it was observed that, in those patients with AMI and chronic severe or moderate periodontitis, 24.2% had HDL-C values lower than 40 mg/dl, 78.8% had LDL-C values higher than 100 mg/dl, 55.2% had triglycerides over 150 mg/dl, and US–CRP over 2 mg/L in 53.3%.

**Conclusions:**

Periodontal disease (moderate, severe, and chronic periodontal disease) may increase the risk of Acute Myocardial Infarction (AMI) by increasing the US-CRP levels.

## Introduction

Periodontitis is a chronic infectious inflammatory disease that occurs commonly in medium age adults, and increases in elderly people. It is not only one of the main causes of dental loss in adults, it has also been considered as a predisposing cause of cardiovascular diseases (CVD).

When expanding the perspective about the natural history of periodontal disease and focusing Periodontitis as a potential exposition itself, an inflammatory response can be identified that exceeds the local level reaching the systemic level [1] and may produce consequences in other organs.

Recently, it has been proposed that Periodontitis may have an etiologic modulatory effect and become a risk factor for several systemic conditions and diseases (pregnancy, diabetes, pre-term birth, low weight birth, pulmonary disease, rheumatoid arthritis and other), especially atherosclerotic vascular disease.

Cardiovascular diseases are the primary factor of premature death in developed and developing countries, and the main risk factors have been identified: hypertension, dyslipidemia, diabetes mellitus, smoking and metabolic syndrome, as some major preventable factors. Others such as age and history of early familiar coronary heart disease are also important.

The pathological basis of coronary heart disease is atherosclerosis, a progressive disease in which there is an increase in intima media thickness as the result of cellular reaction provoked as a response to endothelial injury, mediated by cytokines, growth and pro-inflammatory factors, as well as by periodontal disease, considered as an inflammatory factor (Periodontitis) [2].

The association between Acute Myocardial Infarction (AMI) and Periodontal disease (PD) was suggested by Mattila and colleagues in case-control studies in 1989 and 1993. The authors found an association between periodontal disease, poor oral health and AMI, independent of age, total cholesterol, HDL-C, triglycerides, c-peptide, arterial hypertension, diabetes, smoking, social class, and body mass index (BMI). The hypothesis that oral infections constitute a risk factor for coronary disease, was supported by other study conducted by Mattila et al in 1995, in 214 patients with coronary disease who received dental examination, and found that oral health was a predictive factor of coronary events when age, gender, social status, hypertension, smoking, diabetes, and BMI were controlled [3]. Therefore, the understanding of cardiovascular diseases, periodontal disease (periodontitis) and factors that can associate them is relevant.

In periodontal diseases, local inflammatory response to periodontopathogenic bacteria and their products is characterized by infiltration of inflammatory cells, PMN, macrophages, lymphocytes, and plasma cells, into periodontal tissue [4, 5].

Cytokines are released by active macrophages and in some cases the bacterial challenge response is evident through the liberation of inflammatory mediators such as PGE2, IL1, and TNF-α. These cytokines are involved in periodontal destruction and initiate acute response that includes protein liberation such as C-Reactive Protein (CRP), whose elevation has been identified as a common element to periodontal disease and to cardiovascular diseases [6].

Clinically, CRP seems to be a better indicator for future cardiovascular events than LDL-C [7]. The American Heart Association has recently recommended US-CRP measurement, particularly in subjects with intermediate risk in a global risk determination [8].

Traditionally CRP has been used clinically to monitor infections and immunological disorders [9], as part of an acute non-specific response to many forms of inflammation, infection, or tissue breakdown.

In young adults, the CRP mean concentration is 0.8 mg/ml. Different systemic inflammation markers of cardiovascular diseases have been identified. Special attention has been drawn to CRP levels of 1 - 3 mg/L as a risk factor to cardiac and stroke events. Also, high levels of serum IL-6 have been associated with unstable angina, cardiovascular disease and with other cardiovascular risk factors. Likewise, IL-6 has been established as an inductor of CRP.

Steel et al in 1994 [10] and Bruno G Loos et al in 2000 [11] showed that Periodontitis increases the systemic levels of US-CRP, IL-6 and leukocytes, that lead to an increase of the inflammatory activity in the atherosclerotic lesions, potentially increasing the risk for cardiac and cerebrovascular events [12].

The goal of this study was to determine if US-CRP values associated with periodontal disease are risk markers for AMI and to determine if the US-CRP values associated with recent AMI increase more in patients with periodontal disease.

## Materials and Methods

A case-control study was conducted in 401 adults (30 - 75 years old) who lived in Bogota, Colombia, and attended the Hospital Universitario San Ignacio (HUSI), the Faculty of Dentistry at the Pontificia Universidad Javeriana, and the Fundacion Cardio infantil TAMAMU 1.1® was used to determine sample size (type I error = 0.05; type II error = 0.80, OR 3) and 200 patients were chosen as cases, and 201 as controls. Approvals were obtained from the Research Ethics Committees, and every patient signed the informed consent.

Cases were defined as patients who arrived to the emergency service with AMI, with symptoms of chest pain lasting at least 30 min, ischemic changes in the electrocardiogram, confirmed by specific markers (CK or troponin).

Serum tests (total CK, CK MB, Troponin I, blood count, creatinine, electrolytes, glucose, total cholesterol, triglycerides, HDL and LDL) and electrocardiograms were taken. Once the patient was stable, the informed consent was signed, and after 24 to 48 hours, samples were taken for US-CRP values. For the control group, persons with the same sex and similar ages, without AMI were selected. Complete blood count, blood glucose, total cholesterol, triglycerides, HDL-C, LDL-C and US-CRP were taken. Patients with infections or antibiotic treatment in the last three months were excluded from the study; also those who had received periodontal treatment at least six month before entering the study, and those with ulcers caused by any type of prosthesis, with stomatitis, oral candidiasis, or with less than seven teeth in mouth. The periodontal examination for both groups was performed by three examiners, previously calibrated with the Electronic Florida Probe. Periodontal disease was diagnosed by the presence of bleeding on probing and attachment loss. With these clinical signs the diagnosis was confirmed according to the 1999 Armitage classification. The US-CRP assessment was performed using the IMMULITE method containing one monoclonal and one polyclonal anti-CRP antibodies. This method provides a measurement range from 0.1 to 500 mg/L.

### Statistical analysis

OR and confidence intervals were calculated. Multivariate analysis was performed to determine the association among elevated US-CRP levels, periodontal disease and acute myocardial infarction, adjusting for smoking and other confounding factors identified in the analysis.

## Results

Fifty-six point one percent of the study population (225) were men. The study population mean age was 52.6 years, 18.1% (71) smokers, 31.7% (127) had dyslipidemia, 31.4% (126) hypertension, and 14.2% (57) diabetes (Table 1).

**Table 1:** General Description of the Population

	Total (n = 401)
Men	225 (56.1%)
Age (mean, sd)	52.6 (13.6)
Glycemia (mean, sd)	110.7 (41.3)
Triglycerides > 150 mg/dl	187 (46.6%)
Total Cholesterol (mean, sd)	205 (48.5)
HDL-C < 40 mg/dl	192 (47.9%)
LDL-C > 100 mg/dl	321 (80%)
Diabetes	57 (14.2%)
Dyslipidemia	127 (31.7%)
High Blood Pressure	126 (31.4%)
Smoking	71 (18.1%)
# of teeth	
7 - 10	25 (6.2%)
11 - 20	114 (28.4%)
21 - 32	262 (65.3%)
Carbohydrate Intolerance	76 (18.9%)
US-CRP > 2 mg/L	106 (26.4%)

There were significant differences between the groups in male sex (30.3% in controls vs. 80% in the cases), mean age (47 in the controls and 58 in the cases) and frequency of abnormal US-CRP (0.5 vs. 52%) (Table 2).

**Table 2:** Descriptive Analysis of the Population

	Control (n = 201)	Cases (n = 200)	P value
Men	61 (30.4%)	164 (82%)	< 0.0001
Age (mean, sd)	47 (13.1)	58 (11.5)	< 0.0001
Glycemia (mean, sd)	96.8 (26.2)	124.6 (48.5)	< 0.0001
Triglycerides > 150 mg/dl	79 (39.3%)	108 (54%)	0.003
Total cholesterol (mean, sd)	214.2 (45.8)	195.8 (49.5)	0.0001
HDL-C < 40 mg/dl	141 (70.2%)	51 (25.5%)	< 0.001
LDL-C > 100 mg/dl	164 (81.6%)	157 (78.5%)	0.439
Diabetes	7 (3.5%)	50 (25%)	< 0.0001
Dyslipidemia	39 (19.4%)	88 (44%)	< 0.0001
High Blood Pressure	21 (10.5%)	105 (52.5%)	< 0.0001
Smoking	23 (11.5%)	48 (24.9%)	< 0.0001
# of Teeth			< 0.0001
7 - 10	4 (2%)	21 (10.5%)	
11 - 20	44 (21.9%)	70 (35%)	
21 - 32	153 (76.1%)	109 (54.5%)	
Carbohydrate Intolerance	8 (3.9%)	68 (34%)	< 0.0001
US-CRP > 2 mg/L	1 (0.5%)	105 (52.5%)	< 0.0001
Attachment Loss	130 (64.7%)	179 (89.5)	< 0.0001

The AMI group was divided into one subgroup including 21 healthy persons and those with gingivitis (10.5%), and another subgroup with 179 (89.5%) patients that showed periodontitis. Of the 179 patients with periodontitis, 147 (82.1%) were men with mean age of 59 ± 11.3 years. When groups were compared, the patients with AMI and chronic periodontitis showed HDL-C values below 40 mg/dl in 24.6%, LDL-C values above 100 mg/dl in 78.2%, triglycerides higher than 150 mg/dl in 54.8% and US-CRP higher than 2 mg/L in 52% (Table 3).

**Table 3:** Descriptive Analysis of Patients With AMI, With and Without Periodontal Disease

	AMI (Healthy or Gingivitis)	AMI Periodontitis
	(n = 21)	(n = 179)
Men	17 (81%)	147 (82.1%)
Age (mean, sd)	55 (13.2)	59 (11.3)
Glycemia (mean, sd)	121.86 (30.3)	124.98 (50.3)
Triglycerides > 150 mg/dl	10 (47.6%)	98 (54.8%)
Total Cholesterol (mean, sd)	204 (55.3)	194.83 (48.9)
HDL-C < 40 mg/dl	7 (33.3%)	44 (24.6%)
LDL-C > 100 mg/dl	17 (80.9%)	140 (78.2%)
Diabetes	5 (23.8)	45 (25.1%)
Dislypidemia	10 (47.6)	78 (43.6%)
High Blood Pressure	10 (47.6%)	95 (53.1%)
Smoking	7 (33.3%)	41 (23.8%)
# of teeth		
7 - 10	3 (14.3%)	18 (10.1%)
11 - 20	5 (23.8%)	65 (36.3%)
21 - 32	13 (61.9%)	96 (53.6%)
Carbohydrate Intolerance	9 (42.9%)	59 (33%)
US-CRP > 2 mg/L	12 (57.1%)	93 (52%)

Concerning the periodontal disease breakdown, patients were grouped according to the severity of the disease in healthy, gingivitis and incipient periodontitis in one side, and in the other side moderate and severe periodontitis. Patients with AMI that were periodontally healthy or had gingivitis or incipient periodontitis were just 35 (17.5%), while those with AMI and moderate and severe periodontitis were 165 (82.5%). One hundred and thirty-six of the 165 were men (82.4%) with a mean age of 59 ± 11 years. Comparing the groups it can be observed that those patients with AMI and moderate and severe periodontitis showed lower values of HDL (< 40 mg/dl) in 24.2%, LDL above 100 mg/dl in 78.8%, triglycerides higher than 150 mg/dl in 55.2% and US-CRP higher than 2 mg/L in 53.3% (Table 4).

**Table 4:** Descriptive Analysis of Patients With AMI and Moderate and Severe Periodontitis, and Patients Healthy, With Gingivitis or Incipient Periodontitis

	AMI, and Healthy, Gingivitits, Incipient Periodontitis	AMI and Moderate, Severe Periodontitis
	(n = 35)	(n = 165)
Men	28 (80%)	136 (82.4%)
Age (mean, sd)	55 (13.3)	59 (11)
Glycemia (mean, sd)	125.7 (46.3)	124.4 (49.1)
Triglycerides > 150 mg/dl	17 (48.6%)	91 (55.2%)
Total Cholesterol (mean, sd)	195.6 (47.3)	195.9 (50.1)
HDL-C < 40 mg/dl	11 (31.4%)	40 (24.2%)
LDL-C > 100 mg/dl	27 (77.1%)	130 (78.8%)
Diabetes	6 (17.1%)	44 (26.7%)
Dislypidemia	14 (40%)	74 (44.9%)
High Blood Pressure	17 (48.6%)	88 (53.3%)
Smoking	8 (24.2%)	40 (25%)
# of teeth		
7 - 10	3 (8.6%)	18 (10.9%)
11 - 20	7 (20%)	63 (38.2%)
21 - 32	25 (71.4%)	84 (50.9%)
Carbohydrate Intolerance	17 (48.6%)	51 (30.9%)
US-CRP > 2 mg/L	17 (48.6%)	88 (53.3%)

When the risk of the periodontal disease was evaluated in the AMI group for the US-CRP, it was found that the crude OR was 1.21, with confidence intervals of 0.54 – 2.69. Interactions between moderate and severe periodontitis and the other variables were evaluated, finding out an adjusted OR 1.33, CI 95% (0.59 – 2.98) (Table 5).

**Table 5:** CRP in AMI Patients

	CRP > 2	CRP ≤ 2	TOTAL
Periodontitis (Moderate or Severe)	88	77	165
Healthy, Gingivitis and Incipient Periodontitis	17	18	35
TOTAL	105	95	200

Every patient with Periodontitis (incipient, moderate or severe) was assessed, and the cases were determined as AMI and the control group determined by patients with no history of infarction. Statistically significant differences between groups were found for every variable, with higher levels in AMI patients than in the control group, except for LDL-C > 100 mg/dl which didn’t show differences (Table 6).

**Table 6:** Descriptive Analyisis of Cases and Controls in Patients With Periodontitis

	CONTROLS	CASES	P value
	(n = 130)	(n = 179)	
Men	48 (36.9%)	147 (82.1%)	< 0.0001
Age (mean, sd)	51.2 (10.2)	59 (11.3)	< 0.0001
Glycemia (mean, sd)	99.8 (31.8)	125 (50.3)	< 0.0001
Triglycerides > 150 mg/dl	58 (44.6%)	98 (54.8)	0.079
Total Cholesterol (mean, sd)	220.8 (45.1)	194.8 (48.9)	< 0.0001
HDL-C < 40 mg/dl	83 (63.9%)	44 (24.6%)	< 0.0001
LDL-C > 100 mg/dl	112 (86.2%)	140 (78.2%)	0.076
Diabetes	6 (4.6%)	45 (25.1%)	< 0.0001
Dyslipidemia	26 (20%)	78 (43.6%)	< 0.0001
High Blood Pressure	16 (12.3%)	95 (53.1%)	< 0.0001
Smoking	18 (13.9%)	41 (23.8%)	0.033
# of Teeth			0.005
7 - 10	4 (3.1%)	18 (10.1%)	
11 - 20	35 (26.9%)	65 (36.3%)	
21 - 32	91 (70.0%)	96 (53.6%)	
Carbohydrate Intolerance	6 (4.6%)	59 (33%)	< 0.0001
US-CRP > 2 mg/L	1 (0.8%)	93 (52%)	< 0.0001

When the US-CRP was evaluated for increased risk of infarction in patients with periodontitis (incipient, moderate, severe), a crude OR of 139.5, CI 95% (23.2 – 5609) was found, which shows that having high levels of US-CRP increases the risk of infarction. Given the low number of controls with levels of US-CRP higher than 2 mg/L it was impossible to evaluate the relationship between US-CRP > 2 and other variables. Therefore, when all the variables were controlled, the adjusted OR is 79.6, CI 95% (9.4 – 674.6) (Table 7).

**Table 7:** US-CRP in Patients With and Without Infarction

	Infarction	No infarction	TOTAL
CRP > 2	93	1	94
CRP ≤ 2	86	129	215
TOTAL	179	130	309

## Discussion

According to the European Workshop in Periodontal Health and Cardiovascular Diseases consensus document, there is evidence from epidemiological research on the association between periodontal disease and cardiovascular diseases, although it has been shown as a significant but moderate association [13].

Several studies have established that CRP serum levels and other inflammatory markers (cytokines such as IL-1, IL-6, and TNF-α) are risk factors for coronary disease, although their levels are elevated in both coronary disease and chronic infections. Particularly, Periodontitis increases the systemic levels of CRP, IL-6 and leucocytes, which may increase the inflammatory activity in atherosclerotic lesions and in consequence potentially increase the risk of cardiac and cerebrovascular events [10, 11, 14].

Amol et al, in a meta-analysis published in 2007 [15], on the relationship between periodontal disease and cardiovascular disease after adjusting for confounding factors such as age, gender, cholesterol, diabetes, hypertension, smoking, and body weight, found that both the prevalence and incidence of coronary heart disease are increased with the presence of periodontal disease (periodontitis) and that periodontal disease may be a risk factor for coronary heart disease. This meta-analysis suggests a possible association between periodontal disease and cardiovascular diseases. Higher levels of inflammatory mediators in patients with periodontitis may have a role in the genesis of atherothrombosis, being the rationale for the above-mentioned suggestion.

D’Aiuto et al suggested a possible role of untreated severe Periodontitis on future atherosclerotic process via systemic inflammation. Some studies reported by these authors establish that patients suffering from severe Periodontitis have an increased local production of inflammatory cytokines (IL-1B, TNF-α and IL-6) and a moderate systemic inflammatory response characterized by raised concentrations of CRP, and other mediators [16].

In this study, when patients with AMI were divided into two groups, according to severity of periodontal disease, it was found that patients with AMI who showed periodontal health or gingivitis were only 21 (10.5%), while 179 (89.5%) had periodontitis. A comparison of the groups showed that patients with AMI and periodontitis had higher US-CRP levels above 2 mg/L (52%), suggesting that chronic periodontal disease may be a risk marker for coronary heart disease.

On the other hand, the results presented by Amabile et al in 2008 [17] who also evaluated the relationship between the severity of periodontal disease, inflammatory response and angiographic lesions extent in patients with coronary artery disease (CAD), evidenced the correlation of periodontal disease severity with angiographic extent in coronary lesions, independent of the systemic inflammatory response, although the relation with the last one was also evident. This study suggests that their results support the existence of a direct, independent pathogenic pathway between the two conditions, in which periodontal disease could directly influence CAD by inducing repetitive episodes of bacteremia and enhancing a local inflammatory reaction, according to the statement of Haraszthy et al, 2000.

Danesh et al [14] evaluated markers for inflammation and the presence of antibodies for *Clamydia pneumoniae* and *H. Pylori* in patients with and without coronary disease and found that there wasn’t a causal association consistent in the acute phase of the proteins with seropositivity for *H. Pylori* and IgG for *C. Pneumoniae.* On the contrary, in patients with severe Periodontitis, it has been found that serum levels of CRP, IL-1 and IL-6 are elevated when compared with unaffected populations; this increase in the CRP levels has been associated with high levels of infection associated to periodontal pathogens.

In spite of the statements of several reports (Danesh, Offenbacher, Glurich, Fong, and others) about the infection pathway in the association between PD and CVD, the European consensus in periodontal health and cardiovascular disease addressed that in regard to the systemic effect of the infection burden, the oral bacteria may not correlate with the systemic effect, since their quantity may not be proportional to the systemic exposure to them.

Regardless of the mechanism, systemic inflammation seems to be the basis to explain the nature of the connection between chronic infection and atherosclerosis, in which CRP is a reliable marker of acute phase response to infection or inflammation. Thus, it has been postulated that a moderate increase of the CRP in normal subjects can become a predictor for subsequent coronary events.

Glurich et alin 1998 found that comparing the CRP concentration in healthy patients and patients with periodontal disease and cardiovascular disease, the protein concentration was duplicated in patients with one of the above pathologies, and was 5 fold when both of them were present [18].

This study showed higher US-CRP levels in patients with periodontal disease and previous myocardial infarction than in the group without infarction. In the test group 105 subjects had increased levels, unlike the control group in which only one patient had levels above 2 mg/L.

Analyzing the patients by two groups, on one side the healthy, gingivitis and those with incipient periodontitis, and on the other hand moderate and severe periodontitis, 35 and 165 respectively, revealed that patients with AMI and moderate and severe periodontitis had US-CRP values higher than 2 mg/L in 53.3%. Assessing the risk of periodontal disease in the group of patients with AMI, it was found that the crude OR is 1.21 with confidence intervals of 0.54 – 2.69.

The interactions between the presence of moderate or severe periodontitis and other variables were evaluated and it was found to be not significant, with adjusted OR 1.33, CI 95% (0.59 – 2.98). Therefore, significant results weren’t found although it’s impossible to know whether this is a true negative, or if it’s a false negative, low power, due to the low number of patients in one group.

The above findings are consistent with those reported by Noack et al [6], who observed that the increase of the CRP levels could be associated with Periodontal disease, and found higher levels in patients with moderate and severe periodontitis.

Assessing whether the US-CRP increases the risk of AMI in patients with periodontitis, this study found a crude OR 95% 139.5 (23.2 – 5609) that showed that the higher US-CRP levels increase the risk of AMI. Given the low number of controls (no myocardial infarction) with US-CRP higher than 2mg/L, evaluation of the interaction between the US-CRP > 2 mg/L and the other variables, could not be possible, controlling for all variables the adjusted OR was 79.6, CI 95% (9.4 – 674.6).

In a systematic review, Paraskevas et al in 2008 evidenced that CRP is consistently higher in patients with periodontitis, compared with healthy subjects [19]. As some infectious and inflammatory diseases can be associated with cardiovascular diseases, it is believed that chronically higher levels of CRP in patients with periodontitis aggravate the inflammatory process placed in atherosclerotic lesions, and increase the risk of cardiovascular and cerebrovascular events [20-23].

Regarding to the parameters of the severity of the periodontal disease, there is an evidence of lack of standardization for measuring the attachment loss and the activity of the disease in different studies. Furthermore, the European consensus in periodontal health and cardiovascular disease, questioned about the most appropriate way to measure the periodontal disease, either by destruction (disease’s consequence) or by the exposure itself in terms of infection load or host response. The authors state that the amount of destruction in itself correlates with the increase of the systemic manifestations of the infection, and additionally that in gingivitis the inflammatory lesion is confined to superficial tissue with no deep destruction and very little ulcerated epithelium, different from Periodontitis where in presence of tissue destruction and an ulcerated periodontal pocket, there is a clear association with pro-inflammatory agents. According to these statements of the consensus, in this study, the distribution of the groups was performed based on the assumption of the presence of tissue destruction more than the attachment loss measurement. Therefore, the mild Periodontitis (Armitage classification, Clinical attachment Loss: 1 – 2 mm) were grouped with the gingivitis and the healthy group, with the rationale that in this category there wasn’t enough local destruction able to stimulate the pro-inflammatory mediators.

Since the different methods to assess the severity and the destruction in Periodontal Diseases are very limited, it would be very important to find out the way to assess both the severity and the process of the disease in order to improve the approach to determine the role of the Periodontal disease in the systemic inflammatory process.

In conclusion, this study suggests that periodontal disease (moderate, severe, and chronic periodontal disease) may increase the risk of Acute Myocardial Infarction (AMI) by increasing the US-CRP levels. With the advent of periodontal medicine, the knowledge of the risk factors for the cardiac and cerebrovascular diseases, and the results of this study, continuing the research to establish a strong association between these two conditions and enhancing preventive measures in oral health to improve systemic health are highly recommended.
